# Effect of Lycopene Application in Rats with Experimental Diabetes Using Lipoprotein, Paraoxonase and Cytokines

**DOI:** 10.1007/s00232-013-9575-2

**Published:** 2013-06-19

**Authors:** Sevim Çiftçi Yegin, Fatmagül Yur, Ebubekir Ceylan

**Affiliations:** 1 Health Service Vocational School of Higher Education, University of Giresun, Giresun, Turkey; 2 Department of Biological Chemistry, Faculty of Veterinary Science, University of Yüzüncü Yıl, Van, Turkey; 3 Department of Internal Diseases, Faculty of Veterinary Science, University of Yüzüncü Yıl, Van, Turkey

**Keywords:** Experimental diabetes mellitus, Rat, Lycopene, Lipoprotein, Paraoxonase, Cytokine

## Abstract

This study was conducted with the purpose of researching the effect of lycopene application on lipoprotein, paraoxonase (PON) and cytokines that are projected to be used in the diagnosis and treatment of diabetes by making experimental diabetes. At the end of a 1-month trial period, under ether anesthesia with jelly tubes, blood samples were taken from rat hearts. Blood samples were centrifuged and serum was obtained. From the serum samples, HbA_1c_, paraoxonase activity, lipoprotein levels and cytokines were determined. HbA_1c_ levels and PON activity were found to be *p* < 0.001. At the triglyceride level, with regard to the control group, in all the groups a significant rise occurred (*p* ≤ 0.001). At the cholesterol level, with regard to the control group, a decline was observed in the other groups (*p* < 0.05). At the VLDL level, with regard to the control group, a significant rise was observed in the other groups (*p* < 0.05). At the HDL (*p* < 0.001) and LDL (*p* < 0.05) levels, with regard to the control group, a significant decline was observed in the other groups. At the TNF-α, IL-2, IL-6 and IL-10 levels no difference was found (*p* > 0.05). Experimental diabetes models have an important place in analyzing diabetes complications and determining treatment approaches.

## Introduction

Diabetes is an ever-growing chronic metabolism defect all over the world (Seghrouchni et al. 2002). Lifelong and requiring constant follow up and treatment, diabetes mellitus (DM) decreases the patient’s quality of life considerably because of its acute and chronic complications; it is a chronic, metabolic disease, the morbidity, mortality and economic burden on society of which are high. Deficiency of insulin in target tissue or anomaly of its effect causes disorder in carbon hydrate as well as lipid and protein metabolism (Onat et al. 2006). The other factors involved with the occurrence of the disease are heredity, adiposity, pregnancy, giving birth often, long-duration drug use (diuretic, corticosteroid, etc.), infections, psychological or physical trauma and some pancreatic diseases (Sheweita et al. 2002).

Carotenoids protect human lymphocytes from O^2^ damage and decrease the risks of some diseases and some degenerative disorders including some cancer types. Lycopene has a higher capacity antioxidant effect because of the fact that the β cycle in its structure is opened (Mayne 1996; Miller et al. 1996).

Arylesterases, namely, paraoxonase (PON-1), are calcium ligating ester hydrolases in A group aryldialkylphosphatase-type, high-density lipoproteins (HDLs) according to the system of Aldridge (Antikainen et al. 1996). PON-1 has been studied in the field of toxicology because of its feature of hydrolyzing organophosphate compounds; in recent years it has gained currency by considering that people may be protected from KAH risks because of its antioxidant effect. Serum PON enzyme is an enzyme related to HDL, and it is thought to have an antioxidant function (Erden 2004). Experimental studies have shown that HDL-K of PON-1 is related to Apo-A1 and APO-J (Clustrein) proteins (Abbot et al. 1995; Heijmans et al. 2000; Mackness et al. 1997).

Cytokines are multifunctional polypeptides which are synthesized by different cells in the body; they have important roles in the development of cellular and humoral reactions, the stimulation of inflammatory reactions, the regularization of hematopoietin, the control of the increase and differentiation of cells and the initiation of wound-healing processes (Öztürk 2001).

Cytokines like TNF-α, IL-1 and IL-6 have important effects on the initiation and maintenance of particular chemotaxis and inflammatory reactions. These mediators have meditative and regulatory roles in the progress of diabetic nephropathy and in all complications of diabetes (Navarro et al. 2005).

Experimental diabetes models have an important place in analyzing diabetes complications and determining treatment approaches; in this study, we determined the activities of PON and lipoprotein as well as the cytokine profile to research the effect of lycopene on diabetes.

## Materials and Methods

### Material and Experiment Groups

Animals were provided by University of Yüzüncü Yıl Experimental Animal Unit. Twenty-eight Wistar–Albino rats aged 7–8 weeks were used. Rats weighed 300–350 g at the start of the study. Experimental animals were chosen randomly and categorized into four groups. The first was a control group (*n* = 7), the second was a diabetes group (*n* = 7), the third was a lycopene group (*n* = 7) and the fourth was a diabetes-lycopene group (*n* = 7). Rats were sheltered in cages, where they always had food and fresh water in the 4-week experimental period.

The groups were as follows:

1. *Control group (C)* In seven male rats weighing 300–350 g, with blood sugar levels calculated before the experiment, a 45-mg/kg single dose of physiological serum was injected intraperitoneally (i.p.) Karabay et al. (2006).
2. *Group with diabetes but lycopene was not given (D)* In seven male rats weighing 300–350 g, with blood sugar levels calculated before the experiment, a 45-mg/kg single dose of streptozotocin (STZ) (Sigma, St. Louis, MO) was given in a cold citrate tampon (pH 4.5) i.p. Karabay et al. (2006). After 72 h, glucose levels in the blood samples taken from the tail vein were determined through the Lever Chek-TD-4222 biosensor glucose measuring device and strips. Rats with a blood level >250 mg/dl were diagnosed as having diabetes.
3. *Group with diabetes and given lycopene (D–L)* In seven male rats weighing 300–350 g, with blood sugar levels calculated before the experiment, a 45-mg/kg single dose of STZ was given in a cold citrate tampon (pH 4.5) i.p. After 72 h, glucose levels in the blood samples taken from the tail vein were determined with the Lever Chek-TD-4222 biosensor glucose measuring device and strips. Rats with a blood level >250 mg/dl were diagnosed as having diabetes and given lycopene dissolved in corn oil, 10 mg/kg/day for 28 days by gavage orally.
4. *Group to which lycopene was given (L)* In seven male rats weighing 300–350 g, with blood sugar levels calculated before the experiment, lycopene dissolved in corn oil was given, 10 mg/kg/day for 28 days by gavage orally.

### Biochemical Analysis

For future use in the determination of HbA_1c_, lipoprotein and PON activity and in the analysis of cytokine, lipoprotein serum was put into Eppendorf tubes and frozen at −18 °C until the date of the experiment.

PON enzyme activity was determined using a commercial kit of Rel Assay Diagnostics (Gaziantep, Turkey) by a spectrophotometric method. While the levels of blood HbA_1c_ and blood serum lipoprotein (triglyceride, cholesterol, VLDL, HDL, LDL) were analyzed using a commercial kit (Roche Diagnostics, Mannheim, Germany) with the Hitachi-911 autoanalyzer (Hitachi, Tokyo, Japan), cytokine levels (TNF-α, IL-2, IL-6, IL-10) were analyzed using the platen ELISA commercial kit of e-Bioscience (San Diego, CA).

### Statistical Analysis

All data were analyzed using ANOVA and the SAS program (SAS Institute, Cary, NC).

## Results

The levels of data belonging to the four groups are presented in Table 1.

**Table 1:** Results of PON, HbA_1c_, triglyceride, cholesterol, VLDL, HDL, LDL and cytokine in the DM, DM-lycopene, lycopene and control groups

Group	C	D	DL	L	*p*
	(*n* = 7)	(*n* = 7)	(*n* = 7)	(*n* = 7)	
PON (U/L)	13.06 ± 1.35^bc^	4.61 ± 0.74^c^	16.58 ± 2.12^b^	34.11 ± 3.83^a^	<0.001
HbA_1c_ (%)	1.50 ± 0.04^b^	6.74 ± 0.27^a^	2.38 ± 0.17^b^	3.71 ± 0.10^c^	<0.001
Triglyceride (mg/dl)	60.32 ± 2.85^b^	185.71 ± 32.23^a^	158.77 ± 26.58^a^	188.40 ± 15.20^a^	≤0.001
Cholesterol (mg/dl)	66.00 ± 2.22^a^	53.71 ± 6.77^ab^	61.86 ± 3.35^a^	44.57 ± 2.40^b^	<0.05
VLDL (mg/dl)	12.00 ± 0.58^a^	28.57 ± 4.75^b^	31.71 ± 5.34^b^	28.57 ± 2.63^b^	<0.05
HDL (mg/dl)	31.57 ± 4.80^b^	15.29 ± 2.91^a^	15.00 ± 2.15^a^	8.43 ± 1.17^a^	<0.001
LDL (mg/dl)	22.43 ± 4.52^b^	10.57 ± 1.53^ab^	15.14 ± 5.32^ab^	7.71 ± 0.81^a^	<0.05
TNF-α (pg/ml)	45.66 ± 1.16	44.49 ± 1.01	51.17 ± 5.17	50.80 ± 4.44	>0.05
IL-2 (pg/ml)	400.21 ± 137.62	591.34 ± 106.02	309.17 ± 103.91	503.71 ± 115.04	>0.05
IL-6 (pg/ml)	33.03 ± 0.69	30.50 ± 1.65	38.17 ± 4.30	38.89 ± 4.20	>0.05
IL-10 (pg/ml)	189.04 ± 10.40	248.06 ± 53.69	253.74 ± 23.67	233.30 ± 41.56	>0.05

Values are means ± SEM. Letters signify statistically significant difference

In PON activity, a significant decrease in the diabetes group compared to the control group, a slight increase in the diabetes-lycopene group and a significant increase in the lycopene group were found.

HbA_1c_ levels were the highest in the diabetes group, the value was closer to that of the control group in the diabetes-lycopene group, and this value increased significantly in the lycopene group compared to the control and diabetes-lycopene group.

For triglyceride levels, a statistically significant increase was recorded in the diabetes, diabetes-lycopene and lycopene groups compared to the control group.

Cholesterol levels significantly decreased in the lycopene and the diabetes groups compared to the control group. A statistical result close to that of the control group was recorded in the diabetes-lycopene group.

For VLDL, a significant increase was found in the diabetes, diabetes-lycopene and lycopene groups compared to the control group.

For HDL, a significant decrease was observed in the diabetes, diabetes-lycopene and lycopene groups compared to the control group.

For LDL, a significant decrease in the lycopene, diabetes-lycopene and diabetes groups compared to the control group was observed.

When TNF-α, IL-2, IL-6 and IL-10 levels were analyzed, no significant difference was observed in the other groups compared to the control group.

## Discussion

DM is a metabolic and endocrine disease emerging due to absolute or dependent insulin deficiency or due to insulin resistance and characterized by a carbohydrate lipid and protein metabolism disorder. During the progress of the disease, specific complications such as retinopathy, nephropathy, neuropathy and atherosclerosis occur; and thousands of people die of such complications.

One of the most important among numerous diseases in which changes in antioxidant defense systems are detected is DM. In short, it is a disease which is often encountered in various populations and carries with it many fatal complications.

Among the complications of the disease are hyperglycemia, hyperlipidemia, glucosuria, polyphagia, polydipsia and ketoacidosis; firstly, cardiovascular disorders in the progressive aspects of micro- and macroangiopathies, autonomic neuropathy, retinopathy and nephropathies take place. Many investigations have focused on the important role of oxidant stress in the development of complications (Bell and Hye 1983; Giugliano et al. 1996).

Free radical–originated tissue damage causes pancreatic β-cell dysfunction and prevents the use of glucose in surrounding tissues by decreasing insulin secretion (Evans et al. 2003; Ceriello and Motz 2004). Lycopene, with its antioxidant feature, decreases the damage caused by oxidative stress and lipid, protein and cellular components like DNA by catching free radicals (Agarwal and Rao 2000).

In type 2 diabetes patients, using tomato juice, depending upon the level of plasma lycopene, it has been observed that harmful metabolic products in blood vessels decrease with the oxidation of LDL cholesterol.

Serum carotenoids, among which lycopene exists, are closely related to type 2 diabetes. The levels of glucose metabolism and serum carotenoids depending upon abnormalities of glucose tolerance show a linear decrease (Coyne et al. 2005).

The production of free oxygen radical and lipid peroxide increases, and the defense system is insufficient in diabetes. The increase of free radical production contributes to the onset of diabetes complications and progression. Due to a high glucose level, reactive oxidants causing oxidative damage come out in diabetes. LDL oxidation is the key factor in the development and progression of atherosclerotic lesions. Changes in lipid composition and oxidative stress cause lipid peroxidation in diabetes. On the other hand, serum enzymes which have antioxidant features and are carried with HDL prevent the oxidation of PON LDL and HDL particles. Therefore, analysis of the relationship between oxidation and PON enzyme helps us to reveal the role of PON enzyme in diabetics (Aviram et al. 1999).

PON enzyme has the ability to protect HDL and LDL from oxidation. Various mechanisms have been gaining importance in the explanation of this protective role. It is believed that the enzymes related to HDL (PON1, LCAT, platelet activator factor acetylhydrolase platelet) prevent lipoproteins from oxidative modifications (Mackness et al. 1998). PON protects LDL-K from oxidation which is induced by Cu ion and free radicals (Heinecke 2000). PON1 enzyme found in the HDL-K structure demolishes active lipids in minimally modified LDL, so it may show a protective effect against inflammatory response formation in cells of the artery wall (Nelson and Cox 2000). PON hydrolyzes cholesterol linoleate hydroperoxides in oxidized LDL and specific oxidized phospholipids (Aviram et al. 2000).

In the study, the levels of PON in the diabetes group were significantly low in proportion to the diabetes-lycopene, lycopene and control groups (*p* < 0.001). The PON enzyme level in the lycopene group was the highest in proportion to the other groups. In previous studies decreased PON1 activity in the diabetes group may have occurred due to decreased specific activity or increased oxidant substances because of glycation or an inhibitor in circumvolution, as a result of decreasing of serum concentration (Valabhi et al. 2001; Gürsu and Özdin 2002; Öztürk 2008). Our results support the protective effect of lycopene with the data on PON1 activity.

Peripheral neuropathy caused by a decrease in the capacity of lipid peroxide in diabetes supports the idea that decreasing PON activity is related to increased irritability against arteriosclerosis.

The level of PON-1 decreases in diabetics, in those with KVH, in those who smoke, in hypercholesterolemia, in old age, in obesity, in menopause and in renal insufficiency (Aviram et al. 1999).

In the present study levels of triglyceride were high in the diabetes and other groups in proportion to the control group (*p* < 0.001). The increase was two times higher in proportion to the control group. As Deakin et al. (2005) state in their studies, a positive relationship was determined in the serum PON-1, serum triglycerides, diabetes-lycopene and lycopene groups in our study and a negative relationship was observed between the group of PON-1-activated diabetes and serum triglyceride. The levels of total cholesterol were lower in the diabetes and lycopene group in proportion to the control group (*p* < 0.05). Again, levels of VLDL were higher in the other groups compared to the control group (*p* < 0.05). The quantities of LDL (*p* < 0.05) and HDL (*p* < 0.001) were lower in the other groups compared to the control group. A remarkable result is that the levels of LDL and HDL in the group to which lycopene was given were much lower in proportion to the other groups.

LDL cholesterol may be high or normal in diabetics. Yet, small and dense arteriogenic LDL cholesterol increases. LDL cholesterol >100 mg/dl increases cardiovascular mortality (Mahley et al. 2003).

That VLDL flow toward plasma is faster in type 2 diabetics increases LDL synthesis. VLDL affects LDL since it is a precursor of LDL. Barakat et al. (1990) stated that the cholesterol level in diabetics with normolipidemia and hyperlipidemia and the level of triglyceride which LDL and HDL contain increase and the level of cholesterol decreases. These results are compatible with the results we acquired in our study. Insulin has an effect on HMG-CoA reductase and on the level of triglyceride/cholesterol in the compound of LDL. Insulin deficiency or hyperinsulinemia affects this proportion. In conclusion, LDL uptake decreases and LDL accumulation occurs on the blood vessel wall (Barakat et al. 1990).

Patients with 4086 type 2 DM were observed in the United States in 2002, and the serum level of LDL in 58 % of them was 130 mg/dl or more (Saaddine et al. 2002).

In their study on female mice with nonobese DM, Schloot et al. (2002) asserted that the serum IFN-γ level was low with respect to the control group but that the serum IL-10 level did not change.

Such cytolysis of TNF-α, IL-1 and IL-6 and primarily chemotaxis have a substantial influence on starting the inflammatory response and the continuation of it. These function as mediators and stabilizers in the development of diabetic nephropathy and all complications of diabetes (Navarro et al. 2005). Dalla et al. (2005) found that serum levels of acute-phase markers such as CRP, serum amyloid A, fibrinogen and IL-6 are high.

In their study on female mice with diabetes after 6–8 weeks of STZ, Fidan et al. (2005) found an increase in the level of serum IFN-γ with regard to the control group and that the levels of serum TNF-α, IL-1, IL-2, IL-6 and IL-10 remained stable.

In research done on 85 patients with diabetes and 23 healthy subjects, although serum NO and IL-1β had a synergic effect on the development of diabetes, no difference in the serum levels among the groups was observed; and as a result of this, no assessment can be carried out about the functions of these parameters in diabetic patients just by taking the levels of serum NO and IL-1β into consideration (Yenisey et al. 2001).

Altinova et al. (2006) indicated that in patients with type 1 DM with and without diabetic microvascular complications there was no statistically significant difference among the groups in terms of levels of serum TNF-α and IL-6.

In the study, the role of cytokines on diabetes and the effects of lycopene on the levels of cytokine in the groups with diabetes were examined. TNF-α increased in the lycopene and lycopene-diabetes group with regard to the control group, but this increase was not statistically significant. Additionally, IL-2 was slightly high in the diabetic group with regard to the other groups. Also, there was no difference in the levels of IL-6 among the groups, but IL-10 increased a bit in the other groups, especially in the diabetes-lycopene and diabetes groups in comparison with the control group. But this increase was not statistically important. Similar results on many cytokines in diabetes have been obtained, and it has been concluded that cytokine parameters cannot be used in the evaluation of the pathogenesis of diabetes.

It is considered that the HDL level decreased in the group with diabetes compared to the control group and that the decrease observed in the diabetes-lycopene and diabetes groups might be related to the decrease of cholesterol level in these groups. Again, the level of LDL decreased in the lycopene group in comparison with the other groups.

That the level of cholesterol with lycopene is low in regard to the other groups indicates that lycopene has characteristics that lower the level of cholesterol as well. Because of the cholesterol-lowering feature of lycopene, levels of LDL and HDL were low in the lycopene group.

## Conclusion

The combination of diabetes and lycopene does not have an impact on cytokines. On the other hand, the fact that there is an increase in lycopene in the diabetes-lycopene group and a significant decrease in the diabetes group considering the results obtained from the level of PON enzyme, it can be concluded that these are significant indications that diabetes has oxidative and lycopene has preventive qualities. Considering that oxidative stress has a role in forming diabetes and its complications, such alternative methods as lycopene and supportive treatment approaches should be researched and developed.
